# Cerebrovascular reactivity in multiple sclerosis is restored with reduced inflammation during immunomodulation

**DOI:** 10.1038/s41598-022-19113-8

**Published:** 2022-09-14

**Authors:** Antonio Maria Chiarelli, Alessandro Villani, Daniele Mascali, Nikolaos Petsas, Emma Biondetti, Alessandra Caporale, Anna Digiovanni, Eleonora Agata Grasso, Paola Ajdinaj, Maria D’Apolito, Marianna Gabriella Rispoli, Stefano Sensi, Kevin Murphy, Carlo Pozzilli, Richard G. Wise, Valentina Tomassini

**Affiliations:** 1 Department of Neuroscience, Imaging, and Clinical Sciences, University G. d’Annunzio of Chieti-Pescara, Chieti, Italy; 2 Institute for Advanced Biomedical Technologies (ITAB), University G. d’Annunzio of Chieti-Pescara, Chieti, Italy; 3 Department of Radiology, IRCCS Neuromed, Pozzilli, Italy; 4 MS Centre, Department of Clinical Neurology, SS. Annunziata Hospital, Chieti, Italy; 5 Department of Paediatric, SS. Annunziata Hospital, Chieti, Italy; 6 Brain Research Imaging Centre (CUBRIC), School of Physics, Cardiff University, Cardiff University, Cardiff, UK; 7 Department of Neurology and Psychiatry, Sapienza University of Rome, Rome, Italy; 8 MS Centre, Sant’Andrea Hospital, Rome, Italy; 9 Cardiff University Brain Research Imaging Centre (CUBRIC), School of Psychology, Cardiff University, Cardiff, UK

**Keywords:** Multiple sclerosis, Neurophysiology, Vasodilation, Magnetic resonance imaging, Drug therapy

## Abstract

Cerebrovascular reactivity (CVR) reflects the capacity of the brain’s vasculature to increase blood flow following a vasodilatory stimulus. Reactivity is an essential property of the brain’s blood vessels that maintains nutrient supplies in the face of changing demand. In Multiple Sclerosis (MS), CVR may be diminished with brain inflammation and this may contribute to neurodegeneration. We test the hypothesis that CVR is altered with MS neuroinflammation and that it is restored when inflammation is reduced. Using a breath-hold task during functional Magnetic Resonance Imaging (MRI), we mapped grey matter and white matter CVRs (CVR_GM_ and CVR_WM_, respectively) in 23 young MS patients, eligible for disease modifying therapy, before and during Interferon beta treatment. Inflammatory activity was inferred from the presence of Gadolinium enhancing lesions at MRI. Eighteen age and gender-matched healthy controls (HC) were also assessed. Enhancing lesions were observed in 12 patients at the start of the study and in 3 patients during treatment. Patients had lower pre-treatment CVR_GM_ (*p* = 0.04) and CVR_WM_ (*p* = 0.02) compared to HC. In patients, a lower pre-treatment CVR_GM_ was associated with a lower GM volume (r = 0.60, *p* = 0.003). On-treatment, there was an increase in CVR_GM_ (*p* = 0.02) and CVR_WM_ (*p* = 0.03) that negatively correlated with pre-treatment CVR (GM: *r* = − 0.58, *p* = 0.005; WM: r = − 0.60, *p* = 0.003). CVR increased when enhancing lesions reduced in number (GM: r = − 0.48, p = 0.02, WM: r = − 0.62, p = 0.003). Resolution of inflammation may restore altered cerebrovascular function limiting neurodegeneration in MS. Imaging of cerebrovascular function may thereby inform tissue physiology and improve treatment monitoring.

## Introduction

The brain’s ability to regulate its local blood supply is key to maintaining brain function and tissue integrity. Cerebrovascular dysfunction secondary to neuroinflammation may contribute to the pathophysiology of multiple sclerosis (MS)^1^, through tissue metabolic alterations, subsequent neurodegeneration and ultimately disability progression^2,3^. While reduced energy supply, as assessed by cerebral blood flow (CBF), has been identified in MS in both the grey matter (GM) and the white matter (WM)^4–7^, the effect of neuroinflammation on the capacity of cerebral circulation to adapt blood supply to changing demand remains largely unexplored^8^. This dynamic aspect of vascular function can be investigated through the measurement of cerebrovascular reactivity (CVR), that is, the increase in CBF in response to a vasoactive stimulus, most commonly hypercapnia. The property of reactivity allows blood vessels to regulate blood supply with changes in brain tissue demand for nutrients. Alterations in CVR might be associated with neuroinflammation and neurodegeneration in MS: elevated levels of nitric oxide caused by inflammation may desensitise endothelial and smooth muscle function (vascular habituation), causing decreased vasodilatory capacity^9,10^. This impaired regulation of CBF may lead to a state of tissue hypoxia contributing to damage and degeneration^11^.

Here, we evaluated CVR via blood oxygenation level dependent (BOLD) functional MRI (fMRI) during a breath hold (BH) task (a form of hypercapnic stimulus)^12^ in a cohort of MS patients with no vascular comorbidities. We tested the hypothesis that CVR is reduced in MS, compared to age and gender-matched controls, as a result of inflammation, and that CVR increases with the reduction of inflammation during immunomodulatory therapy. We evaluated CVR in both the GM (CVR_GM_) and the WM (CVR_WM_).

Quantifying alteration of CVR with inflammation and its restoration may inform on novel pathophysiological mechanisms of MS damage and disability, and may lead to the discovery of novel physiological markers of disease status and treatment response.

## Materials and methods

### Participants and study design

**Figure 1:**
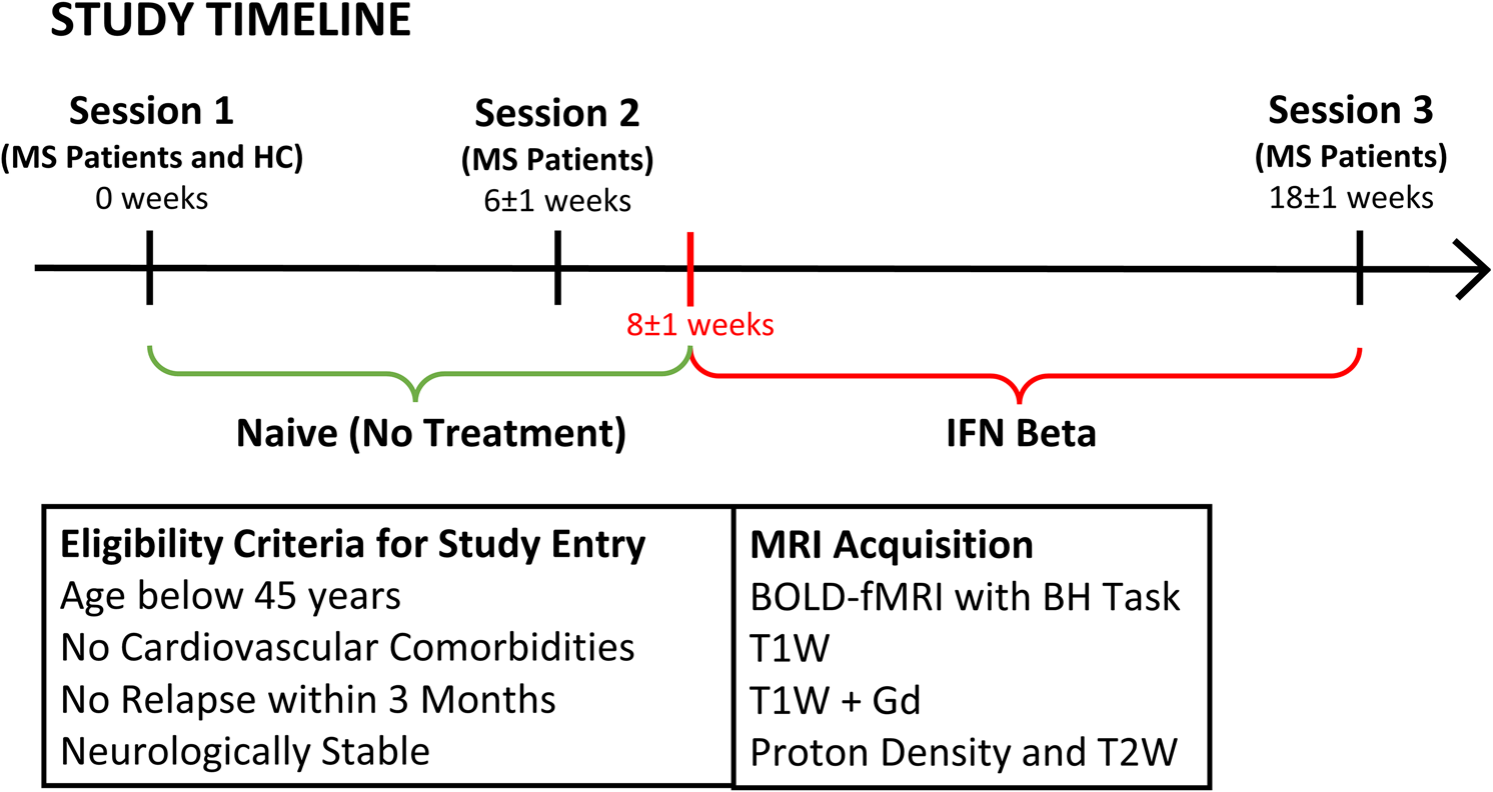
Timeline of the study together with eligibility criteria and MRI sequences performed.

Figure 1 reports information regarding the study participants and design.

Patients with a diagnosis of MS, eligible to start treatment with Interferon beta (IFN beta), were recruited at Sapienza University of Rome, Italy. Patients were included if younger than 45 years, with no relevant comorbidities (including cardiovascular disease) and clinically stable, with no steroid administration and no relapse within 3 months of study entry. Age and sex-matched healthy control (HC) subjects were also recruited. Study participants’ characteristics are reported in Table 1. The study was approved by the Sapienza University Ethics Committee and was performed in accordance with the Declarations of Helsinki. Written informed consent was obtained for each participant prior to the study entry. Patients were scanned twice before treatment initiation [*session* 1 (week 0) and *session* 2 (week 6 ± 1, mean ± SD)] and once (*session* 3, week + 18 ± 1) while on treatment with IFN beta. Expanded Disability Status Scale (EDSS) was assessed in all patients. Behavioural and MRI assessment of age and sex-matched HC was performed once at baseline.

**Table 1:** Demographic and clinical characteristics of MS patients and healthy controls.

	Patients (n = 23)	Controls (n = 18)	*p*
Age	35.6 ± 7.3	32.6 ± 6.5	0.17
Sex (W/M)	18/5	13/5	0.65
Disease duration (months)	21.1 ± 31.7	–	-
EDSS score	1.7 ± 0.7	–	–
Time interval between *session* 2–1 (weeks)	6 ± 1	–	–
Time interval between *treatment start* and *session* 2 (weeks)	2 ± 1	–	–
Time interval between *session* 3–2 (weeks)	12 ± 1	–	–
Overall study duration (weeks)	18 ± 1	–	–
T2 lesion volume at baseline (cm^3^)	2.9 ± 2.7	–	–
Number of Gd+ scans at *session* 1/2/3	12/8/3	–	–

Values are reported as mean ± SD.

### Breath-hold task

**Figure 2:**
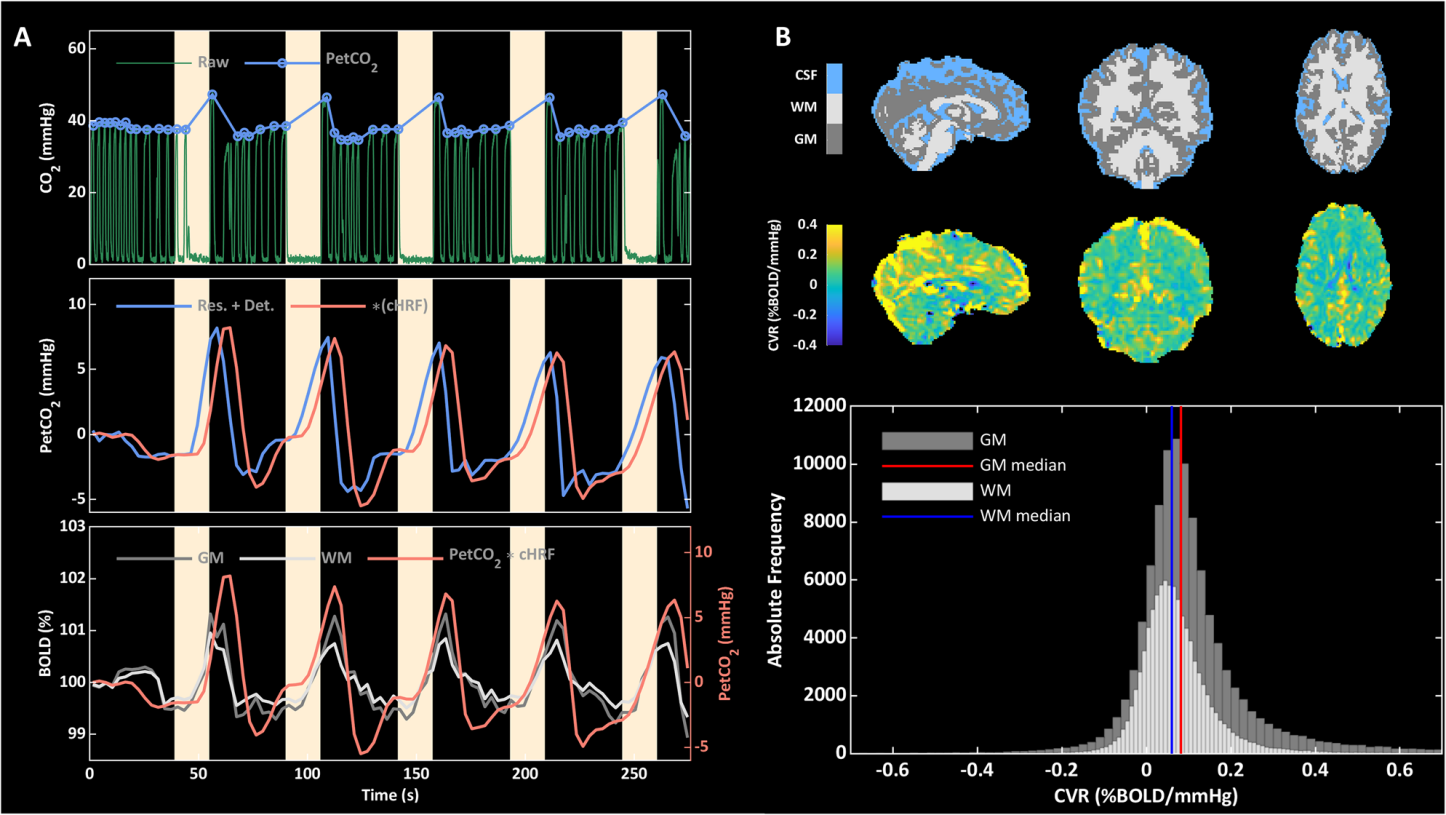
(**A**) Top: Example of raw CO_2_ and PetCO_2_ traces during the breath-hold (BH) task (BH periods highlighted in yellow). Middle: PetCO_2_ trace after resampling, quadratic detrending and convolution with the cHRF. Bottom: Processed PetCO_2_ trace overlaid onto average GM and WM fractional BOLD (%). Processed PetCO_2_ trace was used as a regressor for the voxel-wise GLM analysis. (**B**) Top: Example of brain tissue type segmentation in GM, WM and CSF in one exemplar subject. Middle: Unsmoothed CVR map obtained within the GLM framework in the same subject. Bottom: Voxel-wise distributions of CVR within GM and WM. The median values of such distributions were considered as global estimates of CVRs within the two tissue compartments.

Participants were instructed to perform a repeated breath-hold (BH) task during fMRI following the guidelines reported in the study by Murphy and colleagues^13^. The task comprised five BH repeats each lasting 16 s, followed by a recovery period of 34 s (Fig. 2A). End-expiration BH between paced breathing was employed. In addition, from a position of normal expiration, the participants were asked to expel any residual air from the lungs after the BH periods before returning to paced breathing permitting accurate measurement of changes in end-tidal CO_2_ at the end of the BH. CO_2_ partial pressures were measured in the expired air via a nasal cannula connected to a Medrad Veris 8600 Vital Signs Monitor (Medrad, Pittsburgh, PA, United States). Analogue-to-digital recording of the continuous CO_2_ partial pressure was performed during the MRI using a National Instruments card (NI, Austin, TX, United States). Values of end-tidal CO_2_ partial pressure (PetCO_2_), taken as a surrogate of arterial CO_2_ pressure, were extracted using in-house software in Matlab (Fig. 2A).

### MRI acquisition

MRI images were acquired using a Siemens Magnetom Verio 3 T/70 cm bore magnet (Siemens Healthcare, Siemens, Germany). The list of performed MRI sequences are reported in Fig. 1.

High resolution anatomical T1-weighted scans were acquired for GM and WM volume estimation and for fMRI co-registration (TR = 1900 ms, TE = 2.93 ms, 512 × 512 matrix, FOV 260 × 260 mm, 176 sagittal slices of 1 mm, flip angle 9°). BOLD fMRI data, comprising 92 whole brain T2*-weighted volumes were recorded during the BH task (2D gradient-echo echo-planar imaging, TR = 3000 ms, TE = 30 ms, 64 × 64 matrix, FOV 192 × 192 mm, 50 transverse 3 mm-slices, flip angle 90°). T1-weighted images (TR = 550 ms, TE = 9.80 ms, 320 × 320 matrix, FOV 240 × 240 mm, 25 axial slices of 4 mm, flip angle 9°) were acquired 5 min after the administration of Gadolinium (Gd) to detect the presence of Gd-enhancing lesions (Gd+). Proton density and T2-weighted images (TR = 3320 ms, TE_1_ = 10 ms, TE_2_ = 103 ms, 384 × 384 matrix, FOV = 220 × 220 mm, 25 axial slices of 4 mm thickness with 30% gap, flip angle 90°) were also recorded to evaluate T2 lesion load.

### MRI processing

In MS patients, T2 hyperintense lesion volumes were estimated based on the T2-weighted images at baseline using the region of interest tool in Jim 5.0 (Xinapse System, Leicester, UK; http://www.xinapse.com). For each patient, the resulting T2 ROIs were examined in conjunction with the post-contrast T1-weighted image in order to obtain a manually segmented mask of Gd+ lesions.

T1-weighted structural and T2*-weighted functional MRI scans were processed using open-source functions of FMRIB Software Library v6.0 (FSL, Oxford, UK)^14^. High-resolution structural T1-weighted MRI images were skull-stripped using the Brain Extraction Tool (BET) and probability maps of three different tissue compartments, i.e., cerebrospinal fluid (CSF), WM and GM, were computed using the FMRIB's Automated Segmentation Tool (FAST). Twelve degrees of freedom affine transformations and warping fields registering the structural MRI scans to the MNI152 template were computed, relying on the skull-stripped images, using FMRIB's Linear and Non-linear Image Registration Tools (FLIRT and FNIRT). The transformations were applied to the native and skull-stripped T1-weighted images and to the tissue probability maps. Estimates of WM and GM volumes, normalised to a standard intracranial volume, were calculated by multiplying voxel volume by the sum of the tissue probability maps in the linearly co-registered MNI space. GM and WM masks were established by classifying each voxel as belonging to CSF, WM or GM, based on the tissue compartment with the highest probability. fMRI data were motion corrected (MCFLIRT), expressed as fractional BOLD changes with respect to the temporal means and high pass filtered (gaussian filter, sigma = 60 s) using the *fslmaths* from FSL function. Twelve degrees of freedom affine transformations of the fMRI scans to structural images were computed and BOLD volumes were linearly transformed into the space of the MNI152 template. In addition, twelve degrees of freedom affine transformations of the post-contrast T1-weighted images to structural images were computed and Gd+ lesion masks were linearly transformed into the space of the MNI152 template.

CVR maps, expressed as BOLD signal percent change per unit of PetCO_2_ (%BOLD/mmHg), were estimated by voxel-wise regression of the PetCO_2_ signal with BOLD signal^15^. In detail, quadratically de-trended PetCO_2_ traces, convolved with a canonical hemodynamic response function (cHRF), were used as the independent variables of general linear model (GLM) analysis performed within the FMRI Expert Analysis Tool (FEAT). CVR_GM_ and CVR_WM_ were computed as median values of the GLM β-weights within the GM and WM masks (excluding Gd+ lesion volumes). Also the CVR within the Gd+ lesions was computed as the median value of the GLM β-weights within the Gd+ mask. To perform group-level voxel-wise statistical comparisons of CVR, the β-weight maps in the linearly transformed MNI space were non-linearly warped into MNI, using the already computed non-linear warping fields and spatially smoothed with a low-pass Gaussian filter (sigma = 4 mm).

### Statistical analysis

Kolmogorov–Smirnov test was run to assess if data were normally distributed. Longitudinal effects in the CVR of MS patients were evaluated through one-way repeated analysis of variance rANOVA (three levels, MS *session* 1, MS *session* 2, MS *session* 3) and *post-hoc* paired *t*-tests. Since the longitudinal analysis did not identify differences between the first two sessions, for between-groups comparison with HC, and also for additional longitudinal analysis, an average pre-treatment CVR from the two first sessions was computed for the MS patients. When one of the two pre-treatment sessions was missing, the other was assumed to represent the pre-treatment CVR. Between-groups effects in global CVR were assessed through unpaired *t*-tests. Pairwise associations between variables were evaluated through Pearson's correlation. Comparison of CVR_WM_ and the CVR within the Gd+ lesions was also performed through paired t-tests. Group-level null-hypothesis probability maps of between-groups or longitudinal effects in CVR were computed with the function *Randomize* within FEAT, using the threshold-free cluster enhancement method (TFCE, *p* < 0.05).

## Results

### Clinical information for MS patients

None of the patients received steroids or experienced onset of new symptoms or worsening of previously reported symptoms during the study period. EDSS score was on average low and with a small variability, EDSS = 1.7 ± 0.7, and did not change between the 3 sessions. The average T2 lesion volume was equal to 2.9 ± 2.7 cm^3^ (Table 1).

### Global and voxel-wise CVR

Figure 2B reports an example of a structural brain segmentation and associated CVR map along with the voxel-wise distribution and median value of CVR within the GM and WM masks.

Global GM CVRs (CVR_GM_) and WM CVRs (CVR_WM_) for HC and MS in the three sessions did not show significant departure from a Gaussian distribution (Kolmogorov–Smirnov test: all *p’s* > 0.05). CVR_GM_ and CVR_WM_ were not associated with age in both HC and MS sessions (all *p*’s > 0.05). Moreover, for MS patients in all sessions, CVR_GM_ and CVR_WM_ were not associated with EDSS, disease duration or T2 lesion volume (all *p*’s > 0.05).

**Figure 3:**
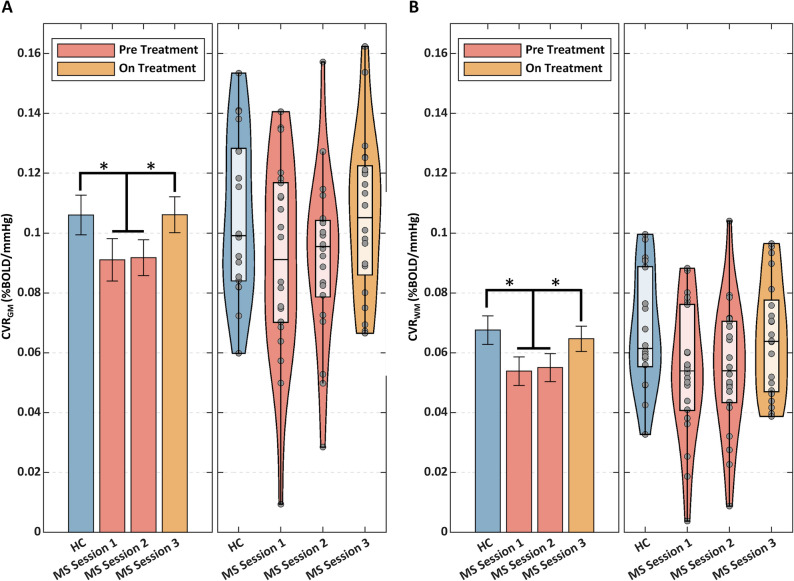
Global average ± SD of the mean CVRs for HC and MS in the three sessions. (**A**) CVR in GM; on the left, mean and associated standard error; on the right, violin plots representing the sample distribution of CVR. (**B**) CVR in WM; on the left, mean and associated standard error; on the right, violin plots. *p < 0.05.

Figure 3 displays bar and violin plots of the global GM and WM CVRs for HC and MS in the three sessions. Barplots report the average CVRs and the associated standard error of the mean for the different groups and tissue compartments (GM and WM in Fig. 3A,B, respectively). Violin plots also report each subject CVR (as dots), together with information about the CVR distribution (minimum, median, maximum value, interquartile range and the expected population distribution). The unpaired *t*-tests highlighted lower CVR_GM_ and CVR_WM_ in pre-treatment MS patients compared to HC (CVR_GM_ = 0.091 ± 0.026%BOLD/mmHg for pre-treatment MS patients, CVR_GM_ = 0.105 ± 0.026%BOLD/mmHg for HC, *p* = 0.04; CVR_WM_ = 0.055 ± 0.020%BOLD/mmHg for pre-treatment MS patients, CVR_WM_ = 0.068 ± 0.020%BOLD/mmHg for HC, *p* = 0.02). For the longitudinal rANOVA analysis of CVR in MS patients, one patient with a missing *session* 2 and two patients with a missing *session* 3 were removed. In patients, rANOVA identified significant differences in CVR_GM_ and CVR_WM_ (both *p’s* = 0.04) between sessions. Paired t-test did not identify differences between *session* 1 and *session* 2, which were averaged. Changes in CVR_GM_ and CVR_WM_ were not associated with age, EDSS, disease duration and T2 lesion volume (all *p*’s > 0.05).

The paired *t*-test revealed an increase in both the CVR_GM_ and CVR_WM_ on-treatment compared to pre-treatment (CVR_GM, On Treatment_ = 0.107 ± 0.027%BOLD/mmHg, CVR_GM, Pre Treatment_ = 0.091 ± 0.026%BOLD/mmHg, ΔCVR_GM, On-Pre Treatment_ = 0.016 ± 0.030%BOLD/mmHg, *p* = 0.02; CVR_WM, On Treatment_ = 0.066 ± 0.019%BOLD/mmHg, CVR_WM, Pre Treatment_ = 0.055 ± 0.020%BOLD/mmHg, ΔCVR_WM_ = 0.011 ± 0.022%BOLD/mmHg, *p* = 0.03).

**Figure 4:**
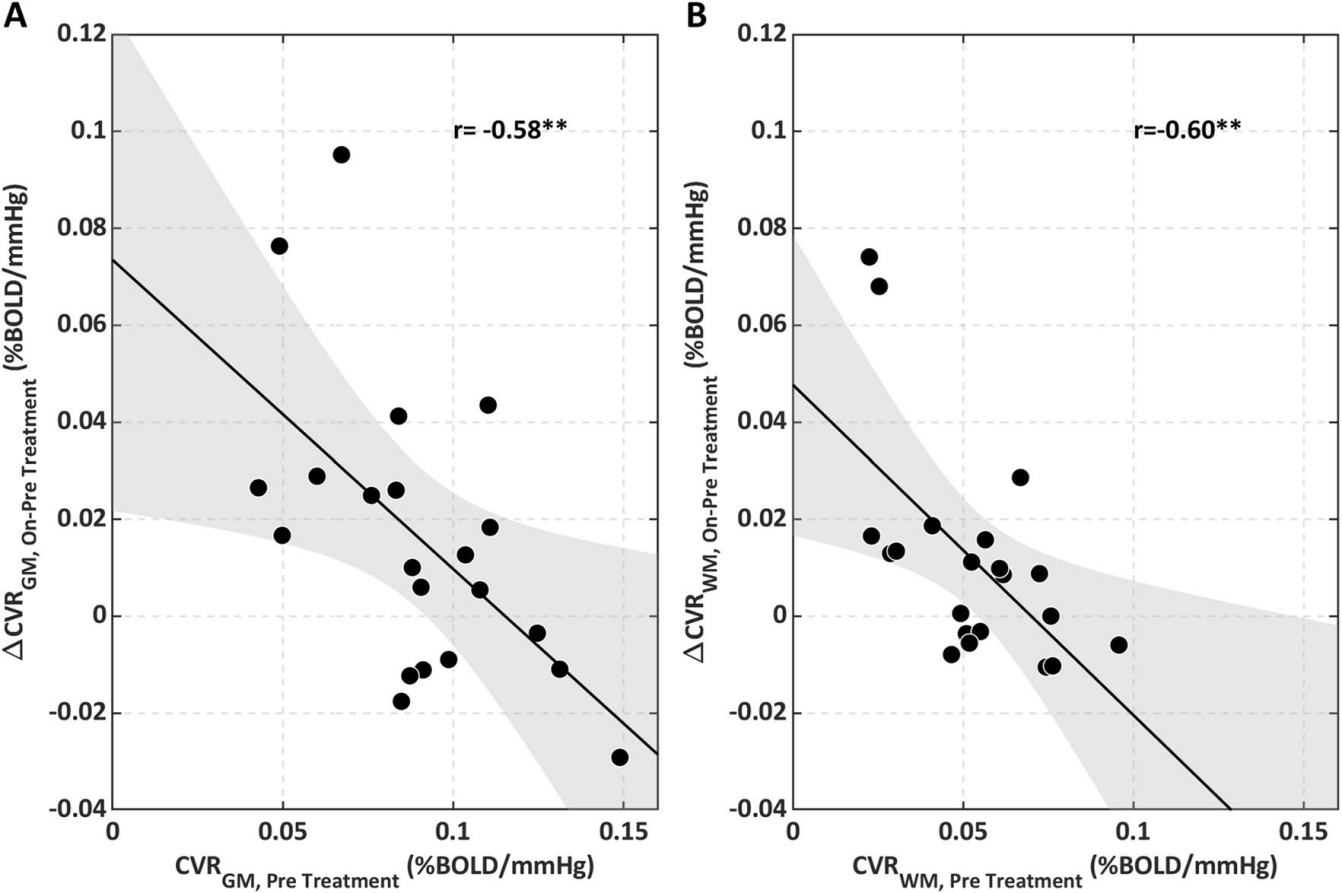
On vs. pre-treatment changes in CVR as a function of the pre-treatment CVR (**A**) in the GM and (**B**) in the WM. **p < 0.01.

Figure 4 reports the magnitude of global CVR changes with MS treatment that was negatively correlated with the pre-treatment CVR in both the GM (*r* = − 0.58, *p* = 0.005, Fig. 4A) and the WM (*r* = − 0.60, *p* = 0.003, Fig. 4B).

**Figure 5:**
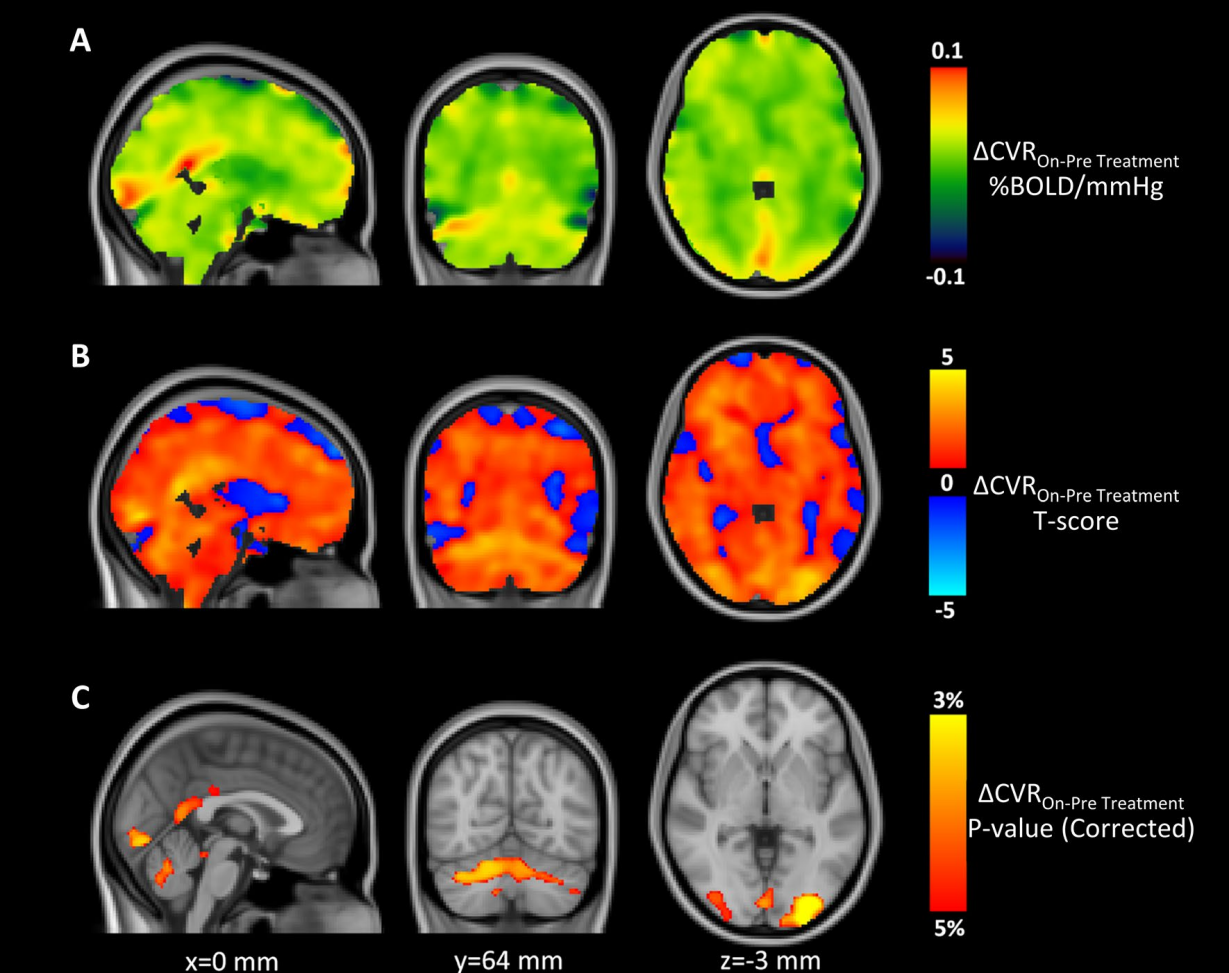
Maps of on vs. pre-treatment change in CVR in MS patients: (**A**) Map in absolute units; (**B**) Statistical Parametric (*t*-score) map; (**C**) thresholded (*p* < 0.05) map of null-hypothesis probability, corrected for multiple comparison using the threshold free cluster enhancement (TFCE) approach. Coordinates (in mm) refers to the MNI152 template.

Figure 5 reports maps of the change in CVR in MS patients while on-treatment. The majority of the voxel-wise changes in CVR and the associated *t*-score were positive, indicating a general increase in BOLD CVR while on-treatment (Fig. 5A,B). Stronger and more significant increases in CVR were seen in the posterior cingulate cortex, in the visual cortex (V1, V2, V3 and V4) and in the cerebellar crus and lobules (primarily, bilateral crus I and II and lobule VI) (Fig. 5C). No significant differences in CVR were observed when comparing voxel-wise MS patients to HC.

### Gd enhancing lesions and global CVR

Twelve, eight and three patients were respectively positive for Gd+ lesions at *sessions* 1, 2 and 3. Patients that were positive for Gd+ lesions had one to five lesions at *sessions* 1 and 2 and one to two lesions at session 3. Qualitative evaluation of the location of Gd+ lesions highlighted that the majority of these lesions were located in the parietal and frontal lobe, followed by the temporal lobe. A minority of lesions were also found in the cerebellum, in the occipital cortex and in the brain stem. No association was found between CVR_GM_ and CVR_WM_ and the number of Gd+ lesions in any given session. However, for the patients with Gd+ lesions, a decreased CVR within the lesions with respect to CVR_WM_ was present for all the sessions and was significant at *session* 1 (CVR_Gd+, Session 1_ = 0.027 ± 0.036%BOLD/mmHg, CVR_WM, Session 1_ = 0.054 ± 0.022%BOLD/mmHg, ΔCVR_Gd+lesions-WM_ = − 0.027 ± 0.030%BOLD/mmHg, p = 0.02).

**Figure 6:**
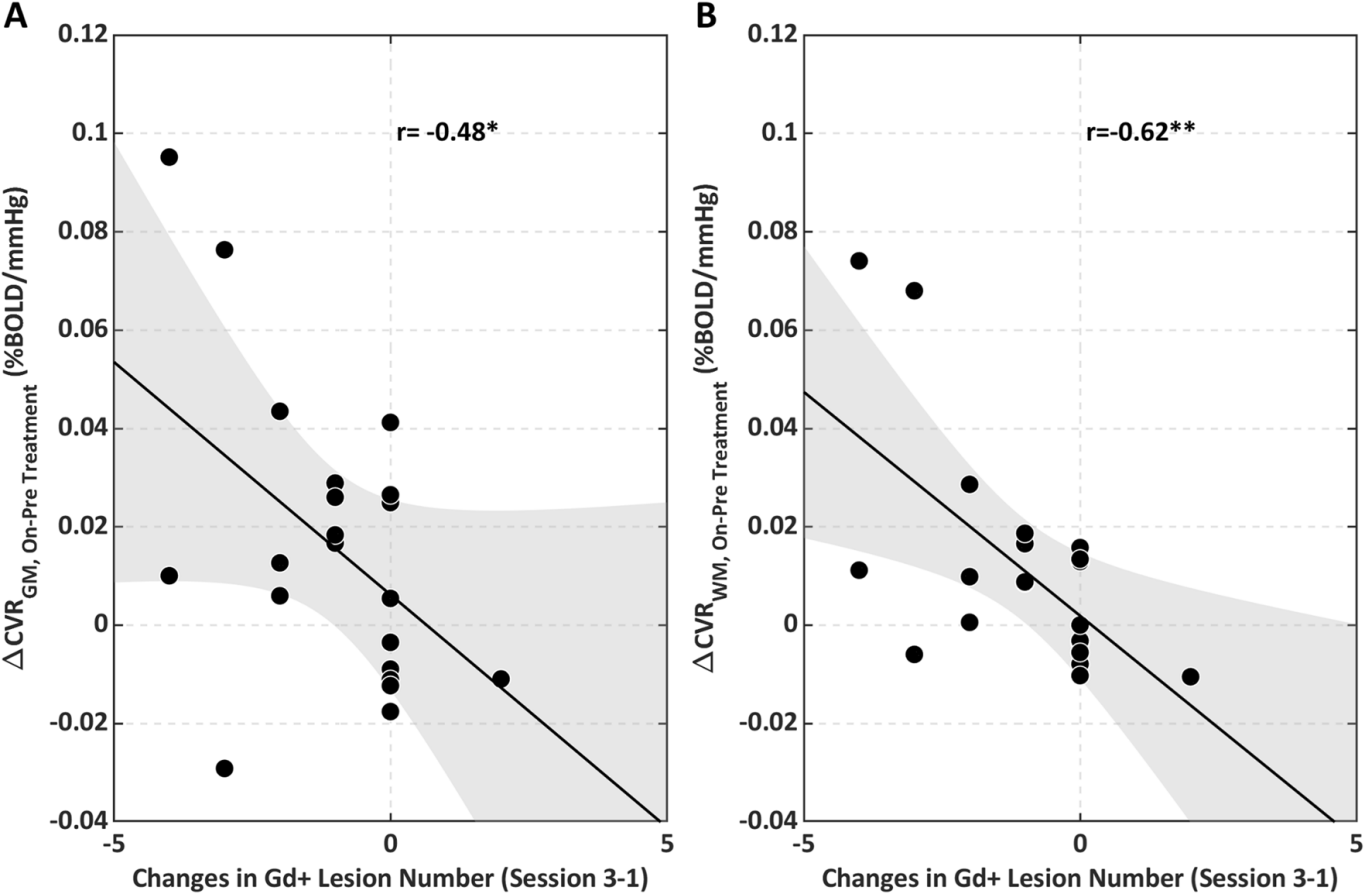
On vs. pre-treatment changes in CVR as a function of the changes in the number of lesions between session *3* and session *1* (**A**) in the GM and (**B**) in the WM. *p < 0.05, **p < 0.01.

Figure 6 reports changes in CVR_GM_ and CVR_WM_ as a function of the longitudinal changes in the number of identified Gd+ lesions from *session* 1 *to session* 3. The CVR_GM_ and CVR_WM_ increases were significantly coupled with a reduction in the number of lesions during the study (CVR_GM_: r = − 0.48, p = 0.02, CVR_WM_: r = − 0.62, p = 0.003).

### GM/WM tissue volumes and global CVR

GM and WM volumetric data (normalised to a standard intracranial volume) were normally distributed (Kolmogorov–Smirnov test: all *p’s* > 0.05). Average GM and WM volumes did not show significant statistical differences between MS patients and HC and among the MS patients for the different sessions (all *p*’s > 0.05). Of note, although not statistically significant, GM volume was higher in HC compared to MS patients for all the three MS sessions.

**Figure 7:**
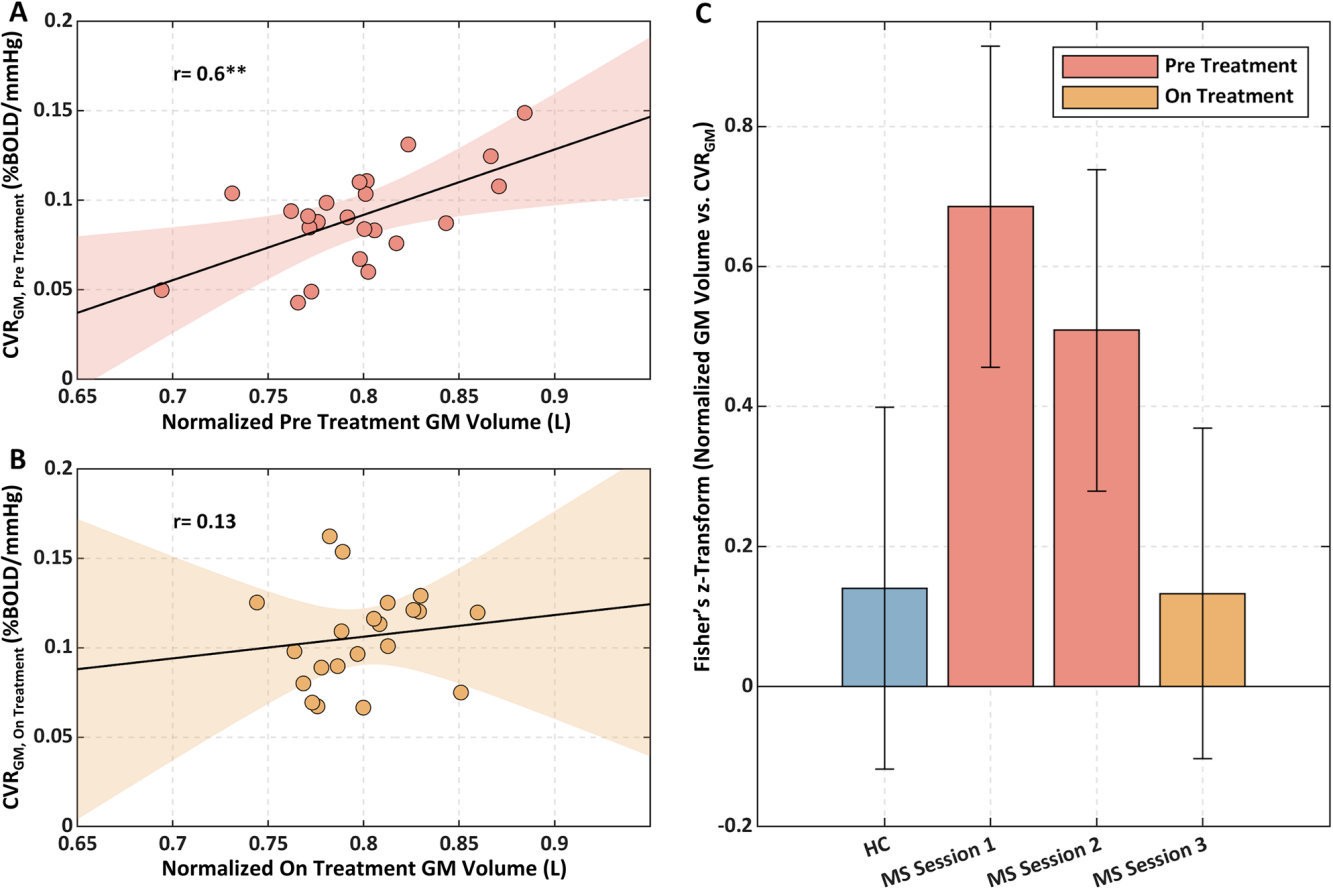
Scatterplots of normalised GM volume *vs.* CVR in GM for MS (**A**) pre-treatment and (**B**) on-treatment; **(C)** Fisher’s z-transforms of the correlations between normalised tissue volumes with CVR in GM and WM for HC and MS patients in the three sessions. **p < 0.01.

Figure 7 reports the correlation of GM volumes with CVR for HC and MS patients (and their estimated SD) in the three sessions. A significant association between tissue volume and CVR was obtained in GM in the patients prior to treatment (r = 0.60, *p* = 0.003) (Fig. 7A). This association was lost with treatment (r = 0.13, *p* = n.s.) (Fig. 7B) and the lack of association was similar to that found in HC (r = 0.12_,_ *p* = n.s.) (Fig. 7C). No significant association was found between CVR_WM_ and WM volume.

## Discussion

Neurodegeneration in MS might be exacerbated by an inability of the cerebrovascular system, affected by inflammation, to maintain a constant nutrient supply^16^. This inability is expected to be causally linked to an altered CVR^10^. CVR in MS, therefore, may offer a relevant marker of disease status, progression and response to treatment. In this study, we demonstrated that a reduction of CVR exists in MS and that it can be, at least partially, reversed by spontaneous or treatment-induced reduction of inflammation.

We found a lower CVR in the GM and WM of MS patients, which were eligible to start treatment with IFN beta, compared to matched HC, a significant lower CVR within the Gd+ lesions with respect to WM and a significant increase in CVR from the pre-treatment to the on-treatment period with IFN beta for both GM and WM which was also coupled with a reduction in the number of Gd+ lesions (Figs. 3, 4, 5, 6). IFN beta acts as an immunomodulatory agent, shifting cytokine networks in favour of an anti-inflammatory pattern^17,18^. CVR changes may reflect restoration of vascular integrity and its hemodynamic mechanisms induced by a reduction of inflammation. Several factors could mediate the association between reduction of inflammation and improvement of cerebrovascular function: oxidative damage resolution, with consequent stabilisation of the blood brain barrier (BBB)^19^; normalisation of endothelial function, with reduction of vasoconstrictive mediators, such as endothelin-1^20^; modulation of vasodilatory mediators^10^. The hypothesis of the study is based on the latter factor: lowering levels of basal nitric oxide, a vasodilatory mediator whose high levels could desensitise endothelial and smooth muscle function (vascular habituation), may restore vasodilatory capacity. The extent of CVR increase in both GM and WM while on-treatment was negatively correlated with the pre-treatment CVR (Fig. 4), supporting the notion of a “restoration effect”, in which greater recovery is associated with a greater initial impairment.

Diffuse increases in CVR were observed over the whole MS brain (Fig. 5), but more significant clusters of positive change in CVR were observed in the posterior cingulate cortex, visual areas and cerebellum, in particular, the crus and lobules. This may reflect a selectively stronger impairment and thus greater recovery in regions more affected by MS, such as the visual cortex and cerebellum^21–23^, or it may reflect a larger sensitivity to BOLD signal changes in regions of greater cerebral blood volume (CBV)^24^. It is unlikely that this finding was related to a larger number of Gd+ lesions in these regions, because lesions were mainly localised to the temporal and parietal lobes.

The spatial and longitudinal coupling between CVR and Gd+ lesions strongly supports the hypothesis of an association of CVR with active lesions and inflammation (Fig. 6). Patients whose MRI scans exhibited a larger reduction in the number of lesions showed a larger increase in CVR. Gd enhancement implies BBB breakdown, typically caused by an inflammatory response and damage due to tissue hypoxia^25,26^: this triggers further amplification of inflammatory processes and cerebrovascular impairment, resulting in leukocyte extravasation and release of cytokines^27^. Restoration of BBB integrity helps to reduce inflammation and to recover the neurovascular unit’s function^28^. Of note, the positive findings were limited to regional and longitudinal effects. A limiting result of the study, probably related to methodological shortcomings, was the absence of significant differences in CVR between patients with and without Gd+ lesions in any session. CVR imaging may therefore selectively complement Gd as a regional marker of brain inflammation or longitudinal marker of its changes.

We observed a pre-treatment association between low CVR and reduced GM volume in MS patients (Fig. 7A,C). Assuming that greater brain atrophy is a marker of neurodegeneration, this result suggests an association of poor cerebrovascular status and inflammation with neurodegeneration. This association was lost while on-treatment. Since GM volume was not modified among the different sessions, this decoupling between CVR and volume in the GM is explained by the greater increase in CVR in patients with the larger initial vascular impairment, i.e., lower pre-treatment CVR (Fig. 4). A strong selectivity of the effect in GM was observed. We hypothesise that, while degeneration of the WM is multifactorial and directly impacted by the dysregulated immune system, neurodegeneration of the GM is, as our study hypothesis suggested, associated with effects of inflammation on the vasculature which may lead to hypoxia-like tissue degeneration^29,30^. In fact, vascular effects may be less pronounced in tissue with diminished vascularization and slower metabolism, such as WM, compared to regions with higher vascularization and higher metabolic activity such as GM.

We did not detect a significant correlation of CVR and CVR changes with age, EDSS, disease duration and T2 lesion volume due to the eligibility criteria of the study that generated a statistically homogeneous cohort of MS patients: all patients had low EDSS score, disease duration and MRI lesion burden with an associated low variability. These criteria were designed to isolate the effects of acute inflammation on CVR, while minimising the influence of factors that could modify cerebral blood flow and hence, CVR^31,32^.

The study was designed to test for the effect of inflammation and its reduction on CVR. For this reason, we included a cohort of patients eligible to start treatment with IFN beta. This cohort was, by definition, inflamed and the changes observed with the introduction of IFN beta could be, at least partially, related to the natural history of MRI active lesions. The reasons for activity reduction, whether spontaneous or drug induced, are not separable in this study.

CVR was assessed through breath-holding, which is easy to implement but it can be affected by biases and diminished repeatability compared to the CVR measured using CO_2_ administration. To limit the shortcomings of this technique, we employed a standardised approach to perform breath holds, and we measured the PetCO_2_ as a surrogate of arterial CO_2_ pressure to account for intersubject variability in task performance. Finally, CVR was assessed in units of %BOLD signal modulation per mmHg of PetCO_2_ change. The change in BOLD signal during hypercapnia is determined by the relative change in CBF that may be altered both through stimulus-induced changes in CBF or by changes in baseline CBF values; these have been reported to be acutely increased in the WM, during active MS phase, but chronically decreased in GM^6,33^. The two factors affecting CVR cannot be decoupled without an independent measure of baseline CBF . The change in BOLD signal is also affected by baseline values of CBV and deoxy-haemoglobin concentration in blood^34,35^. Together with heterogeneous breath-holding performances and subtle temporal differences in the shape of the hemodynamic response following breath holds, the complex origin of the BOLD signal might explain the general robust spatial and longitudinal findings of the study and the weaker between-groups or between-subjects effects. Nonetheless, our CVR maps agreed in range and spatial distribution with previous studies reporting BOLD-CVR maps^36^. With these limitations in mind, our finding of increased BOLD CVR with diminished inflammation might be related to a decrease in baseline CBF, an increase in baseline CBV, or more complex changes in the shape of the hemodynamic response function, rather than an increased vascular responsiveness to the stimulus. These potential mechanisms are relevant as they too highlight the effect of inflammation on brain vasculature.

## Conclusions

Our study suggests an association between MS inflammation and cerebrovascular function linking neuroinflammation with mechanisms contributing to neurodegeneration and thus points towards a target for potential therapeutic approaches. The development of novel physiological MRI approaches linked to vascular function, local oxygen levels and tissue energy status may contribute to monitoring disease states, their evolution, as well as the response to pharmacological treatment in MS. Such an approach may reveal which patients are most vulnerable to vascular and metabolic mechanisms of damage. It may thus enable existing and new treatment strategies to be correctly evaluated in terms of likelihood of physiological recovery, and may contribute to identifying the best treatment strategy in the individual patient, a major goal in precision medicine^37^.

## Data Availability

Data of the present study are available upon reasonable request. Please contact Valentina Tomassini at valentina.tomassini@unich.it.
